# Supplementary figures and images for: Twisting the theory on the origin of human umbilical cord coiling featuring monozygotic twins
Source: Life Sci Alliance. 2024 Jun 3;7(8):e202302543. doi: 10.26508/lsa.202302543 (PMC11147950; doi:10.26508/lsa.202302543)

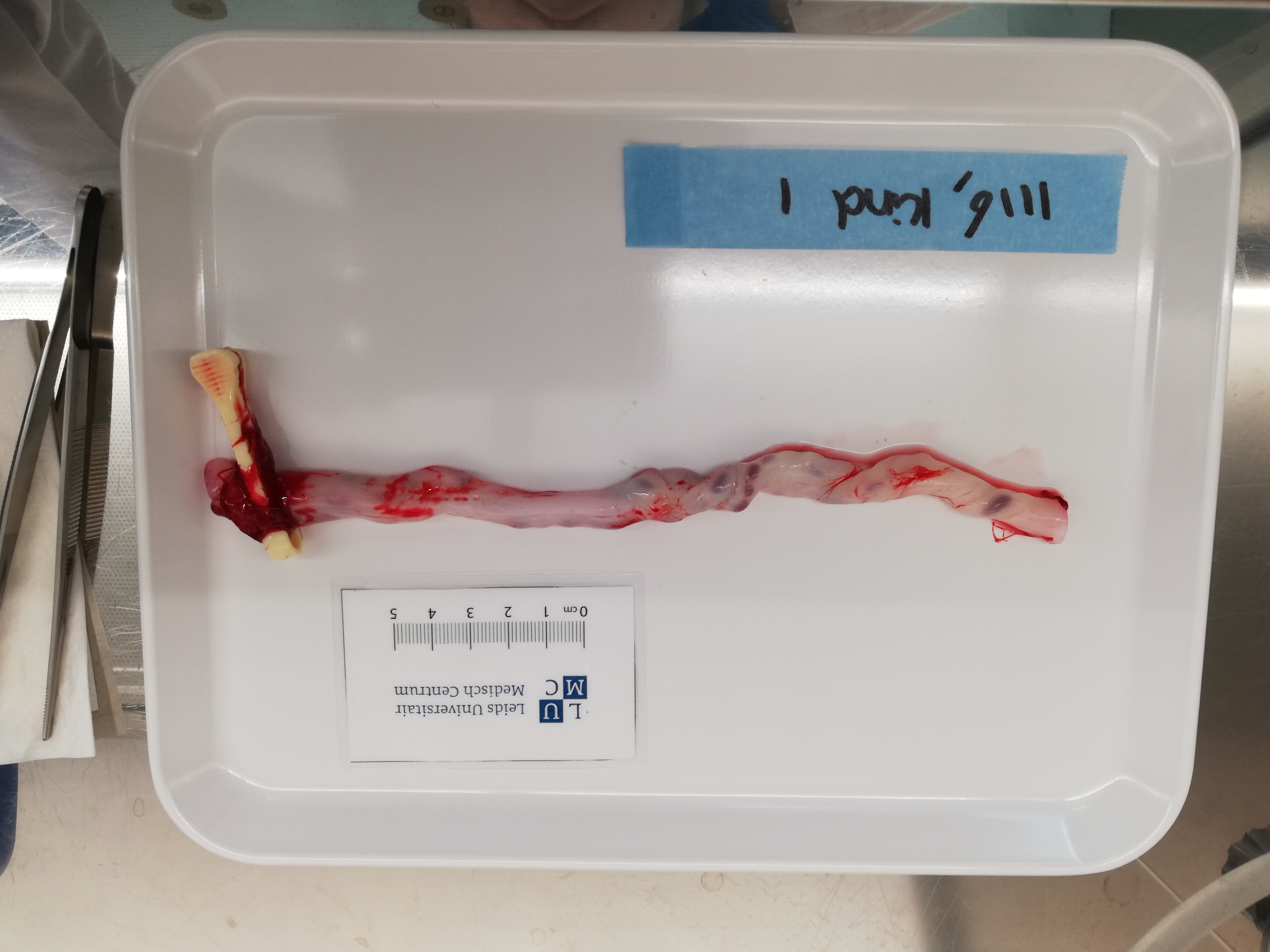

Supplement: Supplementary file 1 [file LSA-2023-02543_SdataF1.1.jpg]

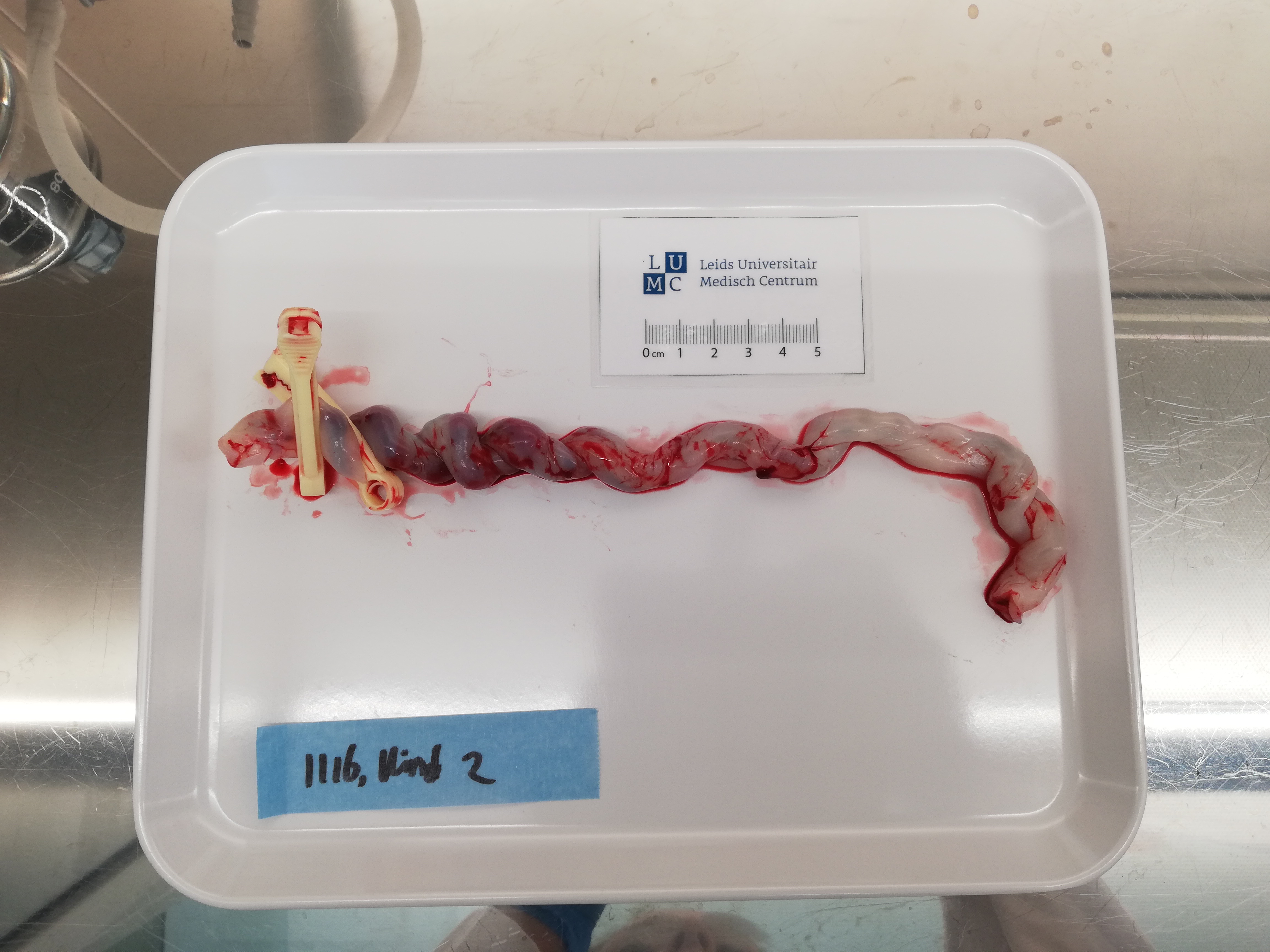

Supplement: Supplementary file 2 [file LSA-2023-02543_SdataF1.2.jpg]

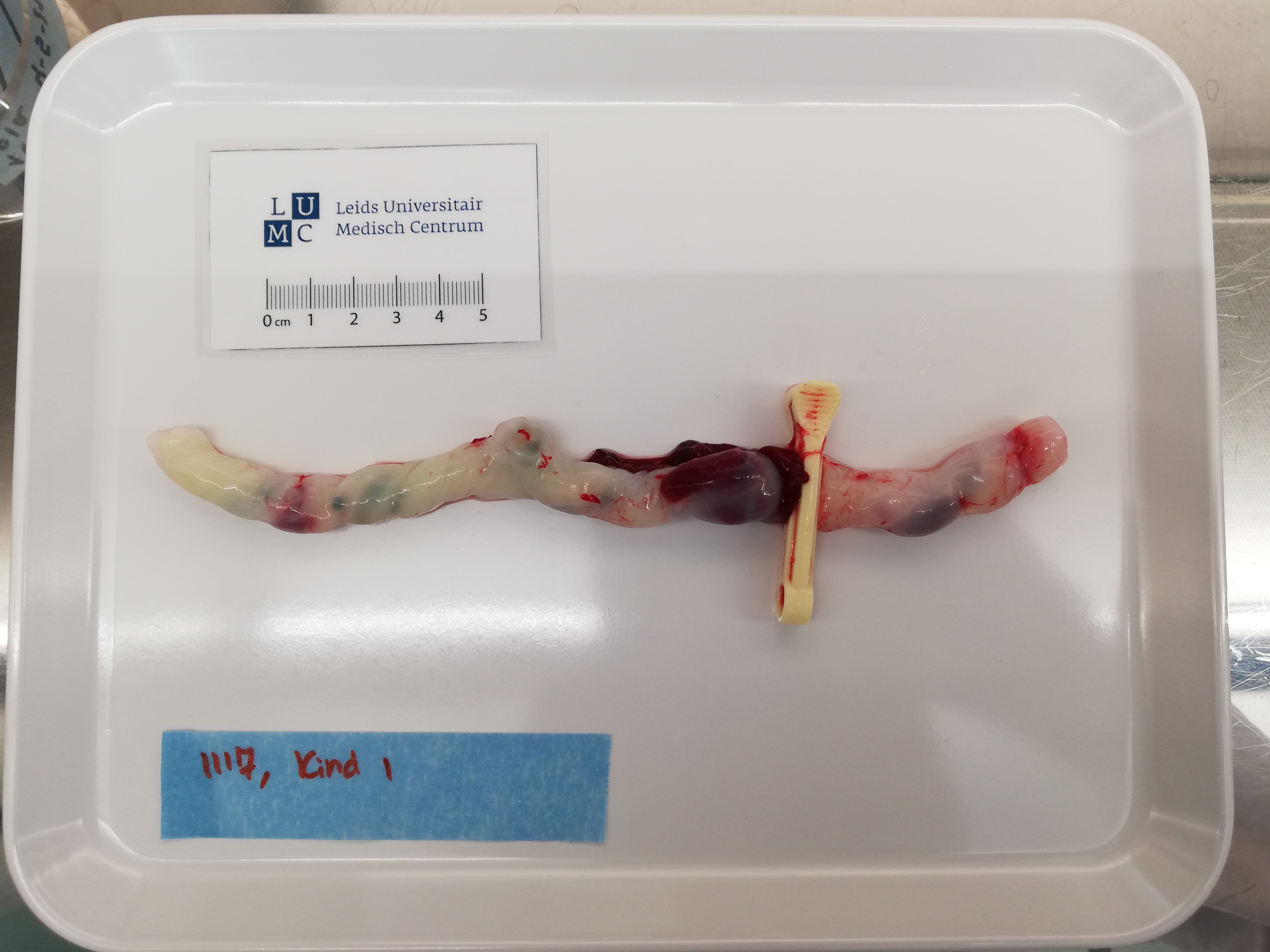

Supplement: Supplementary file 3 [file LSA-2023-02543_SdataF1.3.jpg]

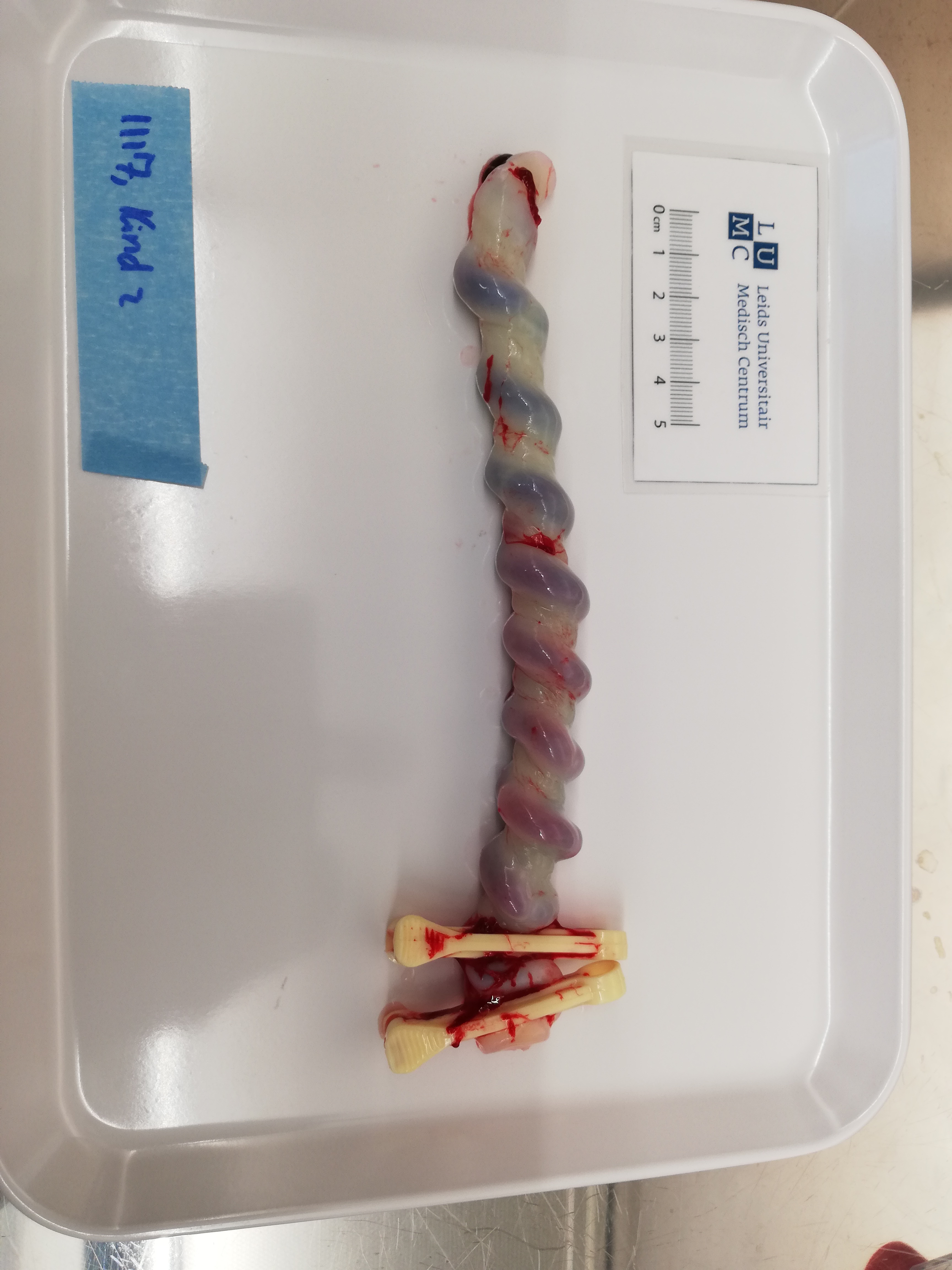

Supplement: Supplementary file 4 [file LSA-2023-02543_SdataF1.4.jpg]

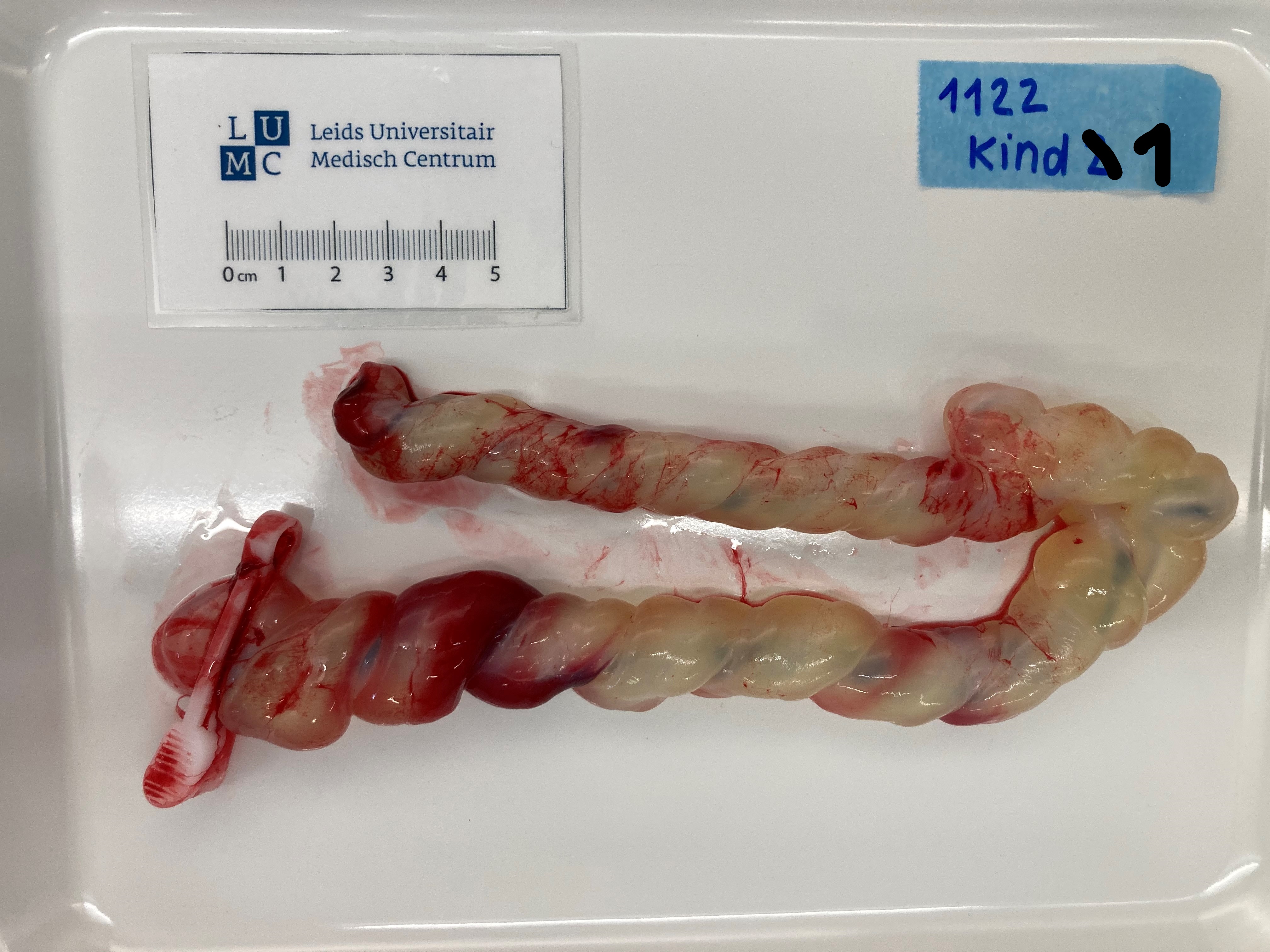

Supplement: Supplementary file 5 [file LSA-2023-02543_SdataF1.5.jpg]

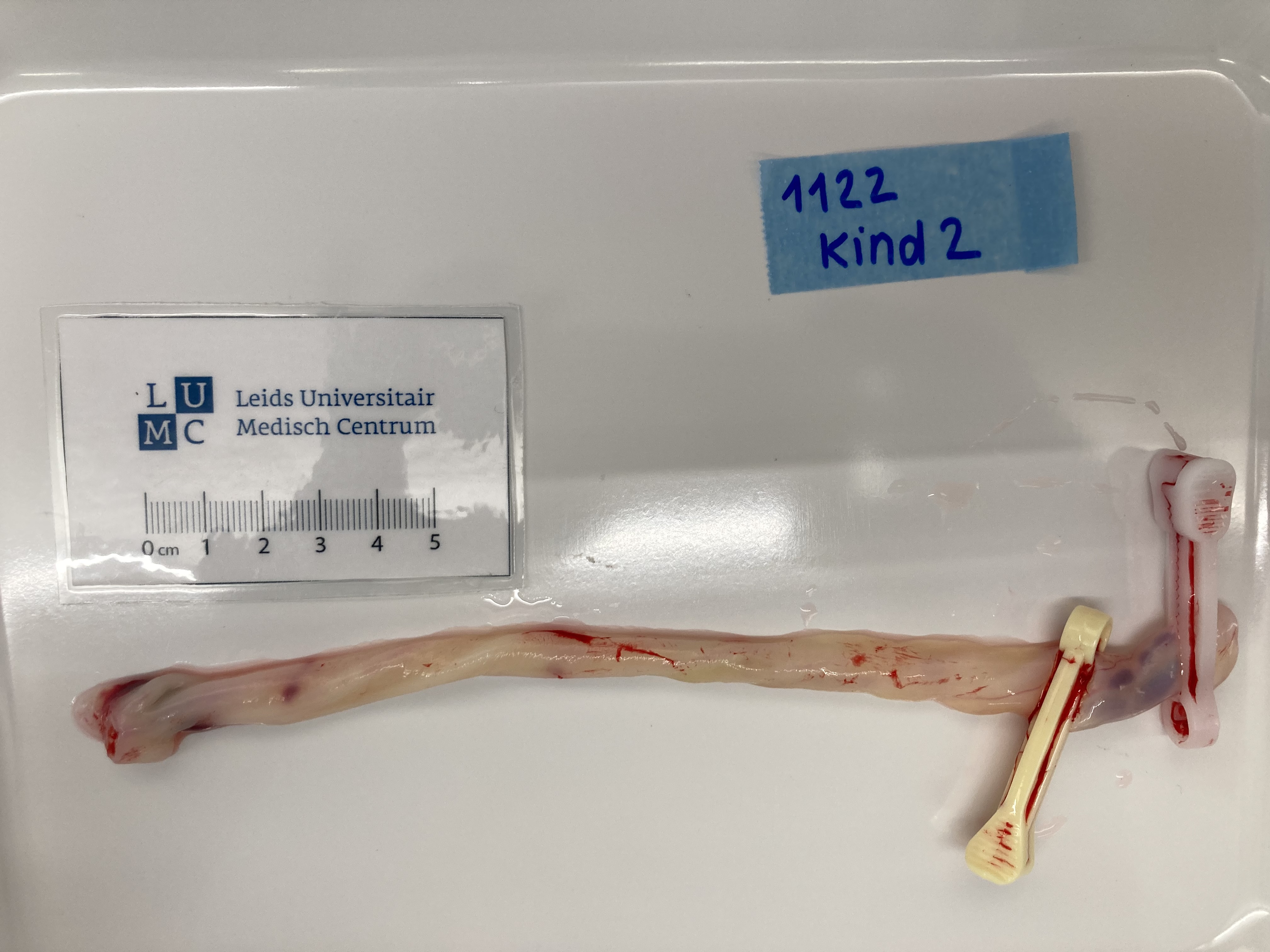

Supplement: Supplementary file 6 [file LSA-2023-02543_SdataF1.6.jpg]

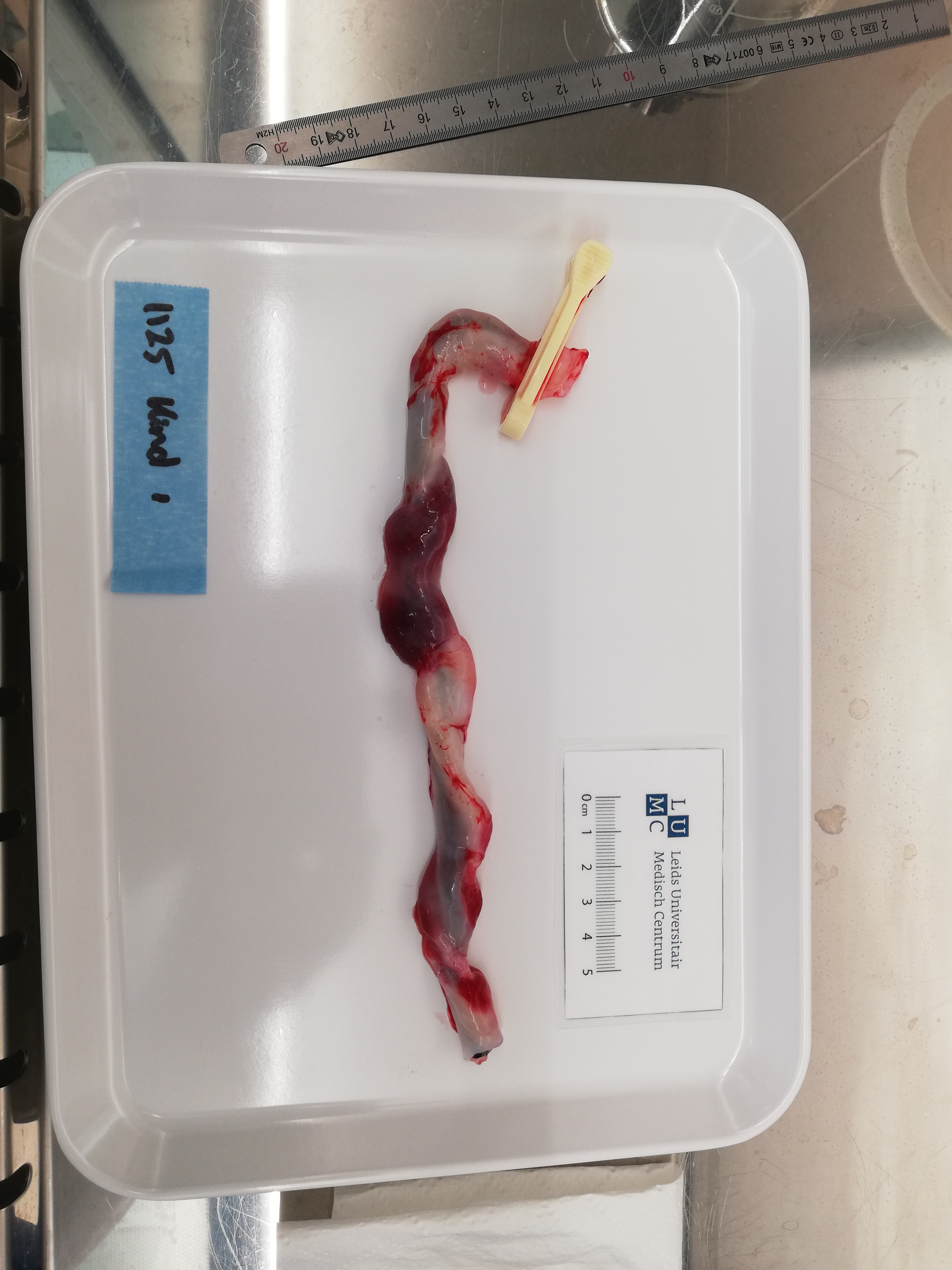

Supplement: Supplementary file 7 [file LSA-2023-02543_SdataF1.7.jpg]

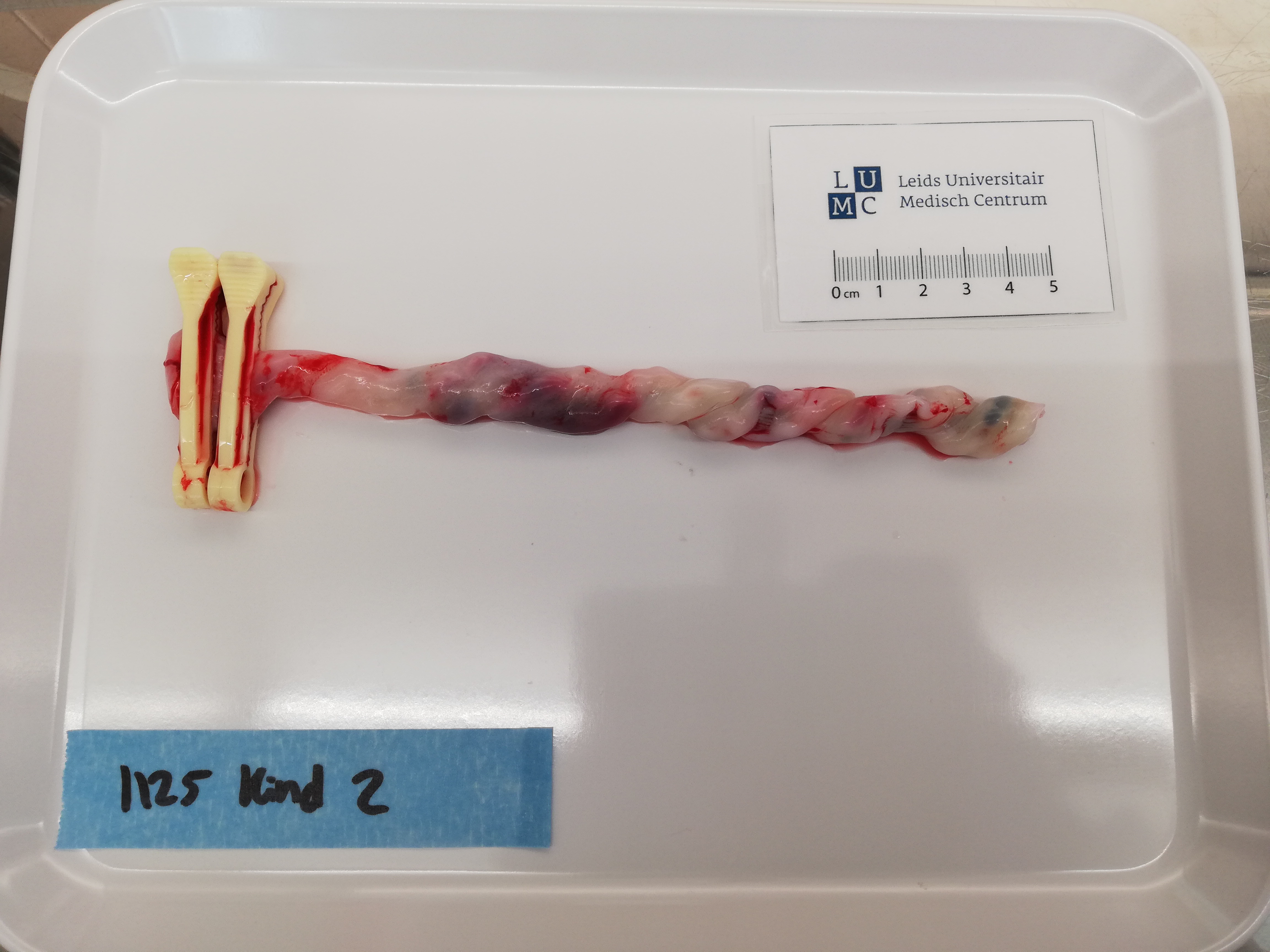

Supplement: Supplementary file 8 [file LSA-2023-02543_SdataF1.8.jpg]

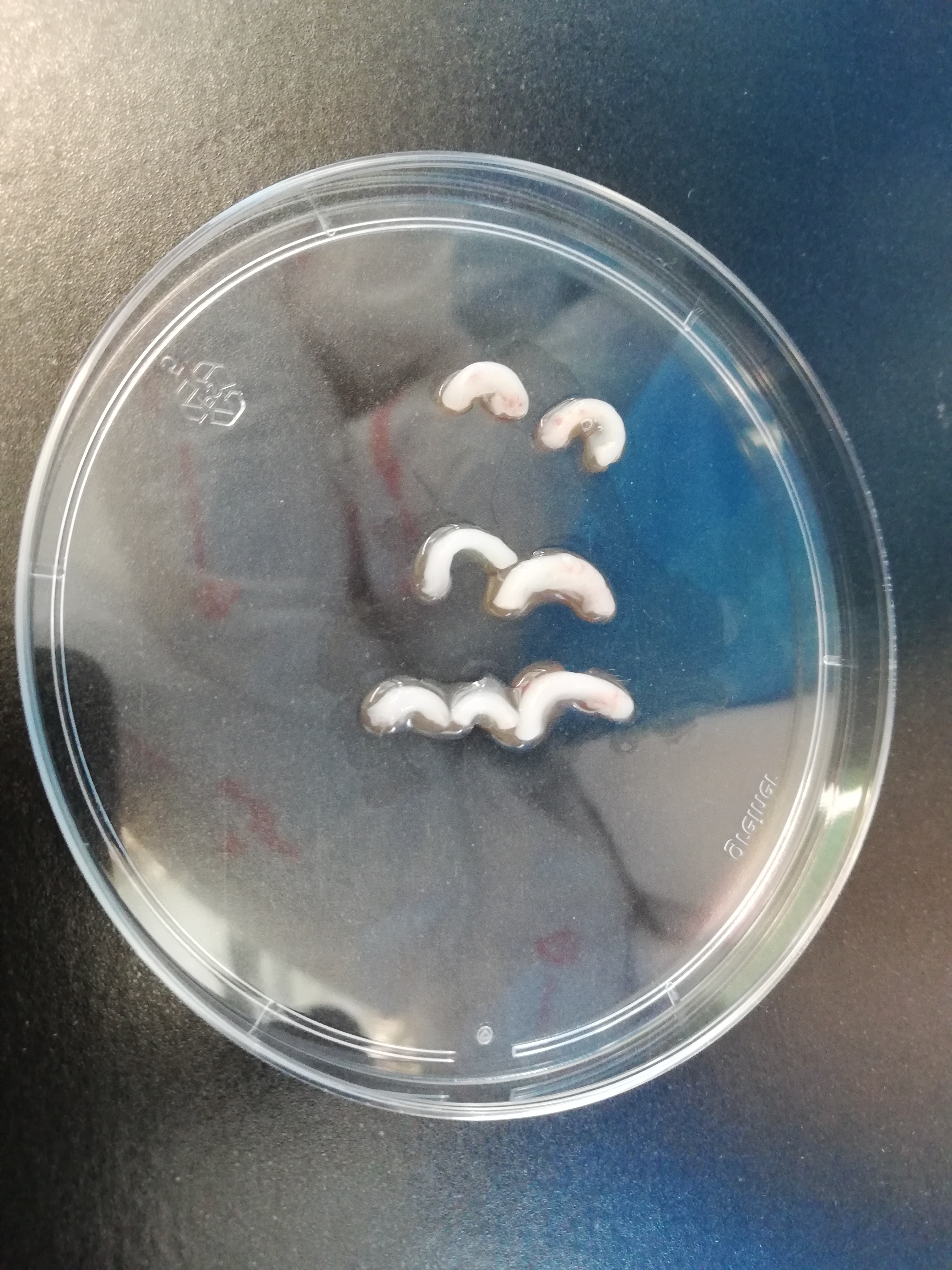

Supplement: Supplementary file 9 [file LSA-2023-02543_SdataF1.9.jpg]

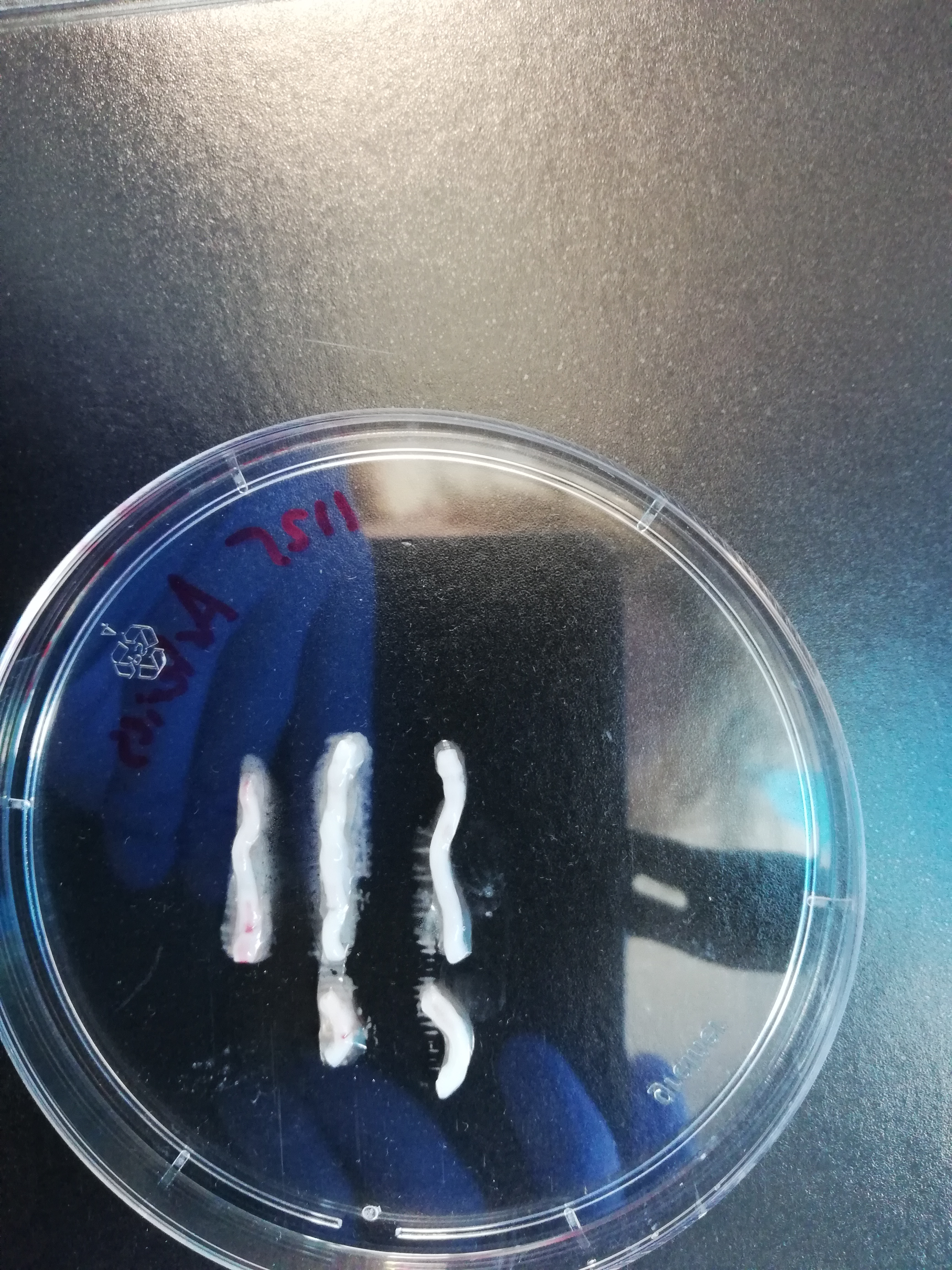

Supplement: Supplementary file 10 [file LSA-2023-02543_SdataF1.10.jpg]

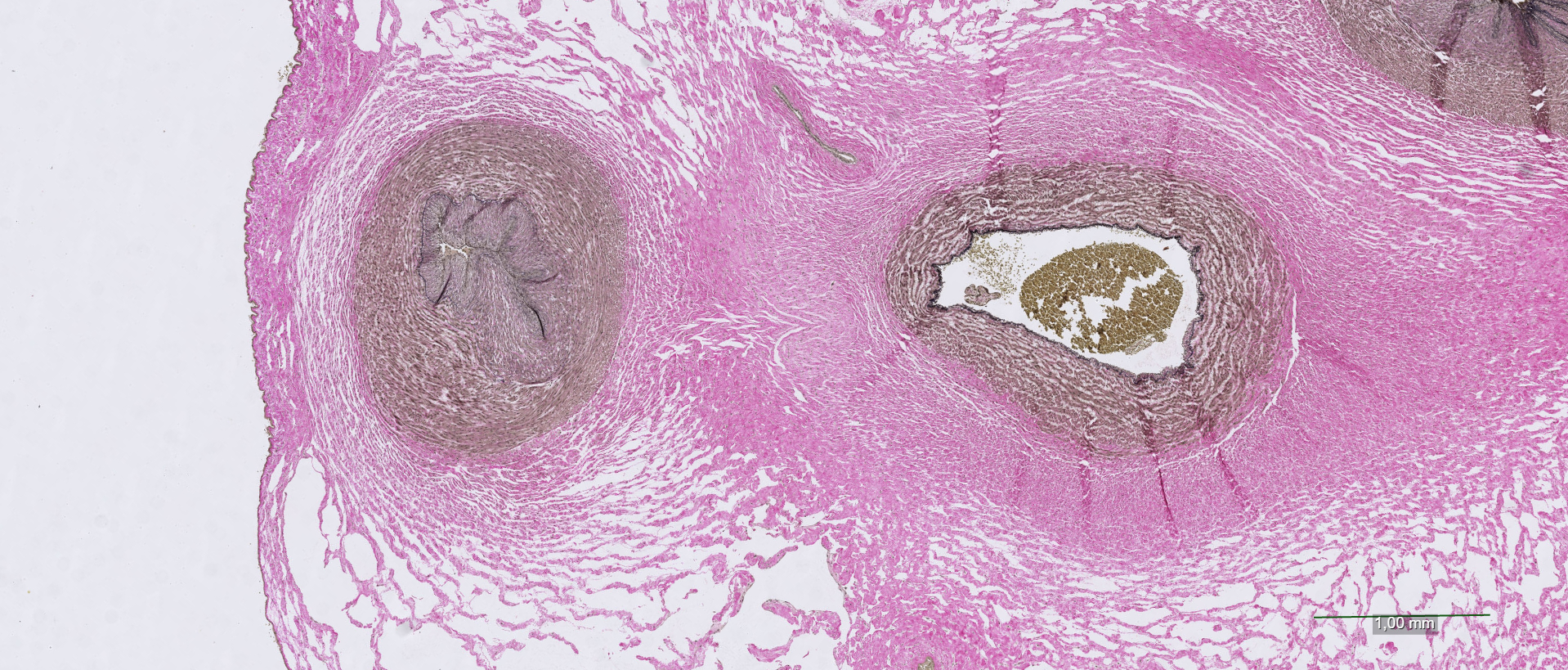

Supplement: Supplementary file 12 [file LSA-2023-02543_SdataF2.1.jpg]

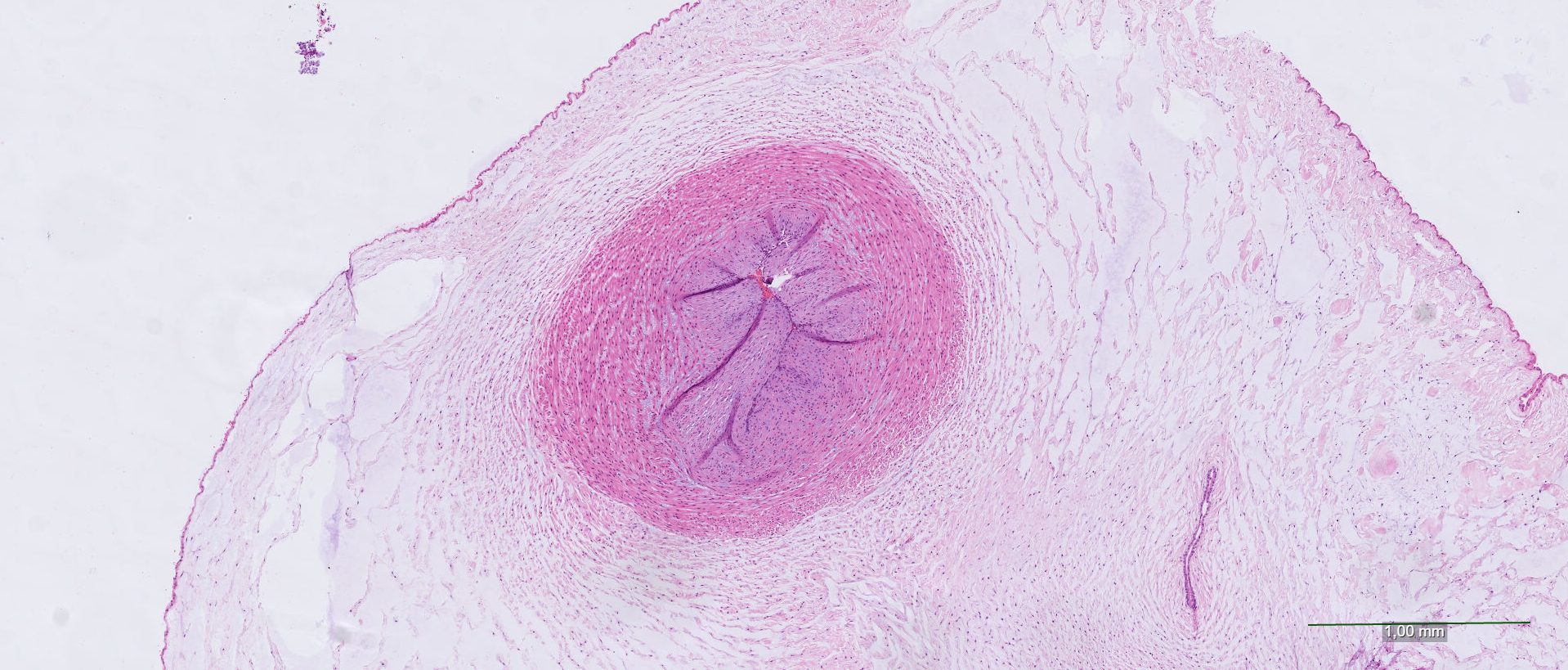

Supplement: Supplementary file 13 [file LSA-2023-02543_SdataF2.2.jpg]

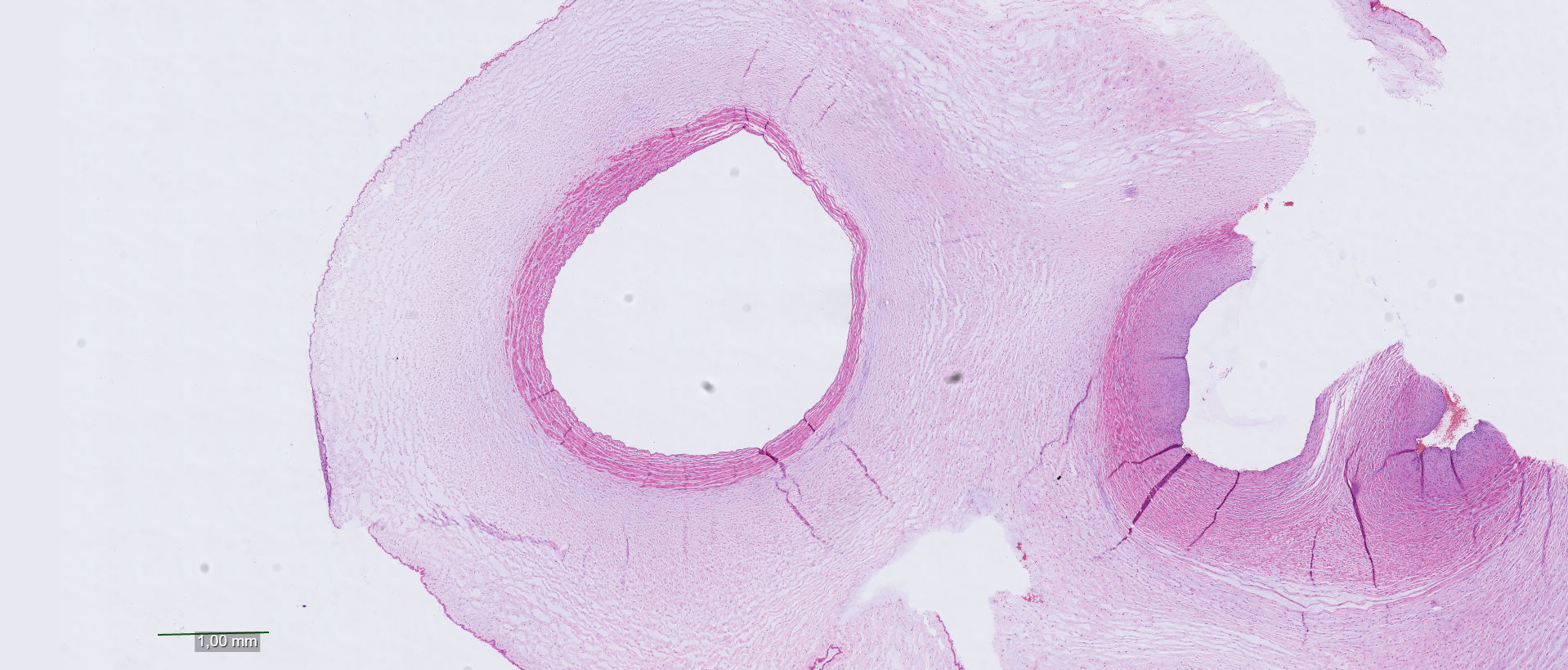

Supplement: Supplementary file 14 [file LSA-2023-02543_SdataF2.3.jpg]
