# Supplementary material for: Twisting the theory on the origin of human umbilical cord coiling featuring monozygotic twins
Source: Life Sci Alliance. 2024 Jun 3;7(8):e202302543. doi: 10.26508/lsa.202302543 (PMC11147950; doi:10.26508/lsa.202302543)
Supplement: Supplementary file 11 [file LSA-2023-02543_TableS1.docx]

**Supplemental Table 1: Donor and umbilical cord characteristics of monozygotic twin pairs discordant for coiling**

| **Case** | **Sex** | **Gestational age**  [weeks] | **Coiling Index**  [#Coils/UC length[cm]] | **Birthweight**  [grams] | **Maternal Age**  [years] | **Caesarean**  **Vaginal Delivery** | **Parity** |
| --- | --- | --- | --- | --- | --- | --- | --- |
| Family 1 Twin 1 | F | 35.6 | 0.19 [4.0/21.0] | 2060 | 27 | Vaginal Delivery | 0 |
| Twin 2 |  |  | 0.30 [7.5/25.0] | 2865 |  |  |  |
| Family 2 Twin 1 | M | 36.3 | 0.29 [6.0/20.5] | 2465 | 35 | Vaginal Delivery | 3 |
| Twin 2 |  |  | 0.45 [6.0/20.5] | 2485 |  |  |  |
| Family 3 Twin 1 | M | 29.0 | 0.42 [18/43.0] | 1836 | 31 | Caesarean | 1 |
| Twin 2 |  |  | 0.09 [2.0/23.0] | 940 |  |  |  |
| Family 4 Twin 1 | F | 32.9 | 0.29 [7.0/24.0] | 1392 | 28 | Vaginal Delivery | 0 |
| Twin 2 |  |  | 0.42 [8.0/19.0] | 1700 |  |  |  |

Characteristics of the umbilical cord donors and the umbilical cord. The umbilical cord donors are eight individuals of four monozygotic twin pairs. When the Coiling Index is >0.3, the cord is categorized as hypercoiled and the genetical identical twin with a UCI <0.3 is categorized as control.
